# Public Health Laboratory Work

**Published:** 1893-12

**Authors:** 


*Public Health Laboratory Work.* By HENRY R. KENWOOD, M.B. Pp. xvii., 491. London: H. K. LEWIS. 1893.

In this volume of "Lewis's Practical Series" the author has collected those processes of chemical analysis relating to water, air, and foods, a knowledge of which is required by the candidate for Public Health diplomas.

The contents, we learn from the preface, broadly represent the course of teaching in the Practical Hygiene Course at University College, and it is undoubtedly a matter of convenience to the student to have collected in one volume those necessary points for which he has hitherto been compelled to search through many text-books. The author has been most successful in dealing with those processes of water analysis of more immediate use to the medical officer of health, and, under the guidance of a practical teacher, the manual should prove a useful guide to laboratory work. Some of the less-used estimations, however, hardly seem to be given with sufficient accuracy and detail, and should be revised in a second edition.

It is doubtfully wise to introduce into a report upon a water so many various ways of stating results as "parts per hundred million," "parts per hundred thousand," and "grains per gallon." Experience generally points to the advantage of adopting a uniform method of notation.

Dr. Rubert Boyce contributes a short but concise summary of methods employed in the bacteriological examination of water, air, and food.

